# Understanding Multicellularity: The Functional Organization of the Intercellular Space

**DOI:** 10.3389/fphys.2019.01170

**Published:** 2019-09-18

**Authors:** Leonardo Bich, Thomas Pradeu, Jean-François Moreau

**Affiliations:** ^1^Department of Logic and Philosophy of Science, IAS-Research Centre for Life, Mind and Society, University of the Basque Country (UPV/EHU), Donostia-San Sebastian, Spain; ^2^ImmunoConcept, CNRS UMR 5164, Bordeaux University, Bordeaux, France; ^3^CNRS UMR8590, Institut d’Histoire et de Philosophie des Sciences et des Techniques, Pantheon-Sorbonne University, Paris, France; ^4^CHU Bordeaux, Bordeaux, France

**Keywords:** control, extracellular matrix, mobility, functional integration, physiology, development, immunity

## Abstract

The aim of this paper is to provide a theoretical framework to understand how multicellular systems realize functionally integrated physiological entities by organizing their intercellular space. From a perspective centered on physiology and integration, biological systems are often characterized as organized in such a way that they realize metabolic self-production and self-maintenance. The existence and activity of their components rely on the network they realize and on the continuous management of the exchange of matter and energy with their environment. One of the virtues of the organismic approach focused on organization is that it can provide an understanding of how biological systems are functionally integrated into coherent wholes. Organismic frameworks have been primarily developed by focusing on unicellular life. Multicellularity, however, presents additional challenges to our understanding of biological systems, related to how cells are capable to live together in higher-order entities, in such a way that some of their features and behaviors are constrained and controlled by the system they realize. Whereas most accounts of multicellularity focus on cell differentiation and increase in size as the main elements to understand biological systems at this level of organization, we argue that these factors are insufficient to provide an understanding of how cells are physically and functionally integrated in a coherent system. In this paper, we provide a new theoretical framework to understand multicellularity, capable to overcome these issues. Our thesis is that one of the fundamental theoretical principles to understand multicellularity, which is missing or underdeveloped in current accounts, is the functional organization of the intercellular space. In our view, the capability to be organized in space plays a central role in this context, as it enables (and allows to exploit all the implications of) cell differentiation and increase in size, and even specialized functions such as immunity. We argue that the extracellular matrix plays a crucial active role in this respect, as an evolutionary ancient and specific (non-cellular) control subsystem that contributes as a key actor to the functional specification of the multicellular space and to modulate cell fate and behavior. We also analyze how multicellular systems exert control upon internal movement and communication. Finally, we show how the organization of space is involved in some of the failures of multicellular organization, such as aging and cancer.

## Introduction

This paper addresses the theoretical issue of functional differentiation, integration, and coordination in multicellular systems. Our claim is that in order to understand multicellularity and its variety of instances, a crucial dimension of this phenomenon has been neglected and needs to be taken into consideration and analyzed: the intercellular space and its functional organization. By developing a theoretical framework focused on the organization of space in multicellular systems, we argue that: (1) cells are not the only and main actors of multicellularity, as highly organized dynamic structures such as the non-cellular ECMs also play a decisive role in the origin and current realizations of functionally integrated multicellular systems; (2) functional spatial differentiation, the control of motility/fixity of cells and the organization of mobility (e.g., vascularization, immune cells, etc.), are three of the main features that allow multicellular systems to overcome bottlenecks of complexity and realize highly integrated and internally differentiated organisms.

Multicellularity is a widespread phenomenon that cuts across all the domains of life, spanning from bacterial biofilms to plants and metazoans. Its most ancient eukaryotic instances date back to around 1.6 billion years ago in the case of red algae (Bengtson et al., 2017). It has been realized independently several times (Bonner, 2000; Grosberg and Strathmann, 2007; Knoll, 2011) including at least one case, *Volvox*, which transitioned to a multicellular form as recently as 200 million years ago (Kirk, 1998). Multicellularity is neither a unique nor a rare phenomenon in the biological word. When it comes to understand it, therefore, it is not a question of explaining its uniqueness, but rather its generality, with deep theoretical implications for our understanding of life in general. It is not by chance, then, that the multicellular dimension of life is at the center of thriving debates in biology and philosophy regarding development, individuality, integration, etc. (Buss, 1987; Arnone and Davidson, 1997; Santelices, 1999; Wilson, 1999; Okasha, 2006; Grosberg and Strathmann, 2007; Michod, 2007; Queller and Strassmann, 2009; Folse and Roughgarden, 2010; Herron et al., 2013; Niklas, 2014).

As argued by several works, the adaptive advantages of realizing multicellular organizations are usually related to increase of size, accompanied by division of labor and increase in complexity, in such a way that multicellular systems can escape predators and occupy different niches with respect to unicellular organisms (Bonner, 2000; Knoll and Bambach, 2000; Rueffler et al., 2012). While doing so, multicellular systems, from biofilms to metazoa, have faced several problems in order to achieve a viable integration between their cellular components. Among the main ones, are the trade-off between cell differentiation and avoidance of conflict, the control and coordination of cells, the availability of nutrients, the access to signal molecules and the possibility of intercellular communication, modularity, structural cohesiveness, to mention the main ones.

To explain how living systems found solutions to these problems, different theoretical approaches emphasize different aspects as the core of multicellularity: self-organization (Newman and Forgacs, 2005), the capability to interpret positional information (Wolpert, 1969), gene regulation (Arnone and Davidson, 1997), cell-to-cell communication (Bonner, 2000; Niklas, 2014) and its role in cell differentiation (Wolpert and Szathmáry, 2002; Arnellos et al., 2013; Veloso, 2017), division of labor between reproductive and vegetative functions (Michod, 2007), genetic homogeneity (Santelices, 1999), low conflict (Queller and Strassmann, 2009), metabolic integration (Pfeiffer and Bonhoeffer, 2003), increase in the energy available and the development of larger genomes (Lane and Martin, 2010), among others.

Yet, as pointed out by Grosberg and Strathmann, multicellularity is not a problem of principle, insofar as many of the requirements that have been suggested for multicellularity – such as cell-adhesion, communication, differentiation, coordination, etc. – have already evolved in unicellular organisms (Grosberg and Strathmann, 2007, see also Brunet and King, 2017). That should not be a surprise, given the importance of the social/communitarian dimension of life (Shapiro, 1988), which has inspired even possible scenarios on origins of life as emerging from colonies of protocells (Woese, 2002; Carrara et al., 2012; Mansy, 2017). The question, thus, is not as much to explain how and why multicellularity originated, as it is of understanding what is specific of multicellular organizations, and how to account for the different forms they can achieve.

The question, then, is what is so special about the interactions that take place in a multicellular system that is not already realized by unicellular organisms. In order to find an answer, it is necessary to understand how cells are *controlled* or *constrained* in their living together in multicellular systems in such a way that they realize and maintain viable organized entities. When these forms of control fail, or their properties change in certain ways, this change may give rise to different (transient or stable) forms of multicellular organization or regressions, more often incompatible with the original one, such as in cancer (Sonnenschein and Soto, 1999; Bissell and Radisky, 2001; Soto and Sonnenschein, 2011) and aging (Moreau et al., 2017).

Our thesis is that in order to understand how cells are constrained and integrated in higher order systems and how several structural and organizational bottlenecks are overcome, looking at cells and their interactions is not enough. We argue that the debate on multicellularity has actually been driven by an implicit cellular bias, so that some fundamental features of multicellular organization have been overlooked by a perspective that identifies in cells the main and only actors of multicellularity. We show that multicellular forms of life cannot be explained exclusively in terms of cellular interactions and their biochemical mechanisms. Rather, we argue that in order to provide a theoretical framework to understand multicellularity, it is necessary to also take into account a dimension that is missing or underdeveloped in current accounts, that is, the intercellular space. By that we mean not only considering the space in which cells operate, and how they specify it, but also how the organization of space, in turn, has a direct influence on cell fate and behavior. It is our contention that the increase in size which characterizes multicellular organisms, and which enables cell differentiation and division of labor, goes hand in hand with and directly depends for its viability on the capability to organize the intercellular space.

Multicellular systems, in fact, are not just made of cells, but of highly dynamical and active structures such as extracellular matrixes (ECMs), which do not just provide structural support for cells, but give rise to a variety of inherently organized intercellular spaces. The importance of space, form, and physical constraints in general has been stressed in the past, but in this paper we will develop a different and more specific point, i.e.: that the organization of space plays a functional role, and the non-cellular structures involved are to be considered as actors of multicellularity together with cells. We will show that the functional properties related to space contribute to many of the features that are considered as fundamental in the debate on multicellularity and that the dynamic nature of space organization has relevance for development and robustness. How the intercellular space is organized is crucial in the control of the fate and activity of groups of cells, in the differentiation of functionally distinct areas, in providing nutrients and enabling communication, ensuring protection, etc. In addition to that, the increase in overall size, accompanied by the loss of the capability of motility in the majority of the cells of a multicellular system, requires the reorganization of mobility and realization of distinct communication subsystems (i.e., the vascular system, the immune system, and the nervous system).

Despite its crucial role at the multicellular level, the organization of the intercellular space has not been the object of a sufficient attention in the literature. Therefore it requires a comprehensive theoretical account capable to bring together and to make sense of the different contributions from biology and medicine, and to inspire further research. This paper aims to do so by developing a theoretical framework that takes into account at its core the question of the organization of space and can contribute to a better understanding of multicellular phenomena. Moreover, we argue that in order to understand the organization of space, the roles of ECM and of mobility and communication systems need to be analyzed. In particular, ECM structures act as control subsystems through mechanical as well as molecular interactions. They play a crucial functional role in maintaining systems of cells viably together, and are instrumental in the transition from unicellular to multicellular systems, as it is shown in the case of *Volvox carteri* (Kirk, 2005). Moreover, not only the organization of space is crucial to understand the realization and viability of multicellular systems in all domains of life, but also different ways of organizing space can account for the distinctive features of different multicellular forms, such as biofilms and eukaryotic multicellular systems, from *Volvox carteri* to metazoa.

The paper will proceed as follows. In Section “Why Multicellular Systems Are Not Just Balls of Cells: The Limits of Current Accounts of Multicellularity,” we analyze the main conceptual issues related to multicellularity and how different theoretical approaches address them. We show that these accounts exhibit some deep conceptual problems, and we argue that they derive from the fact that they do not directly tackle, at their foundations, questions regarding the organization of space. In Section “The Functional Organization of Space,” we introduce and develop the idea of functional organization of the intercellular space by analyzing the role of extracellular structures as control subsystems, and by specifying their functional contribution to the integration of multicellular systems. In Section “Motility, Mobility, and Communication Within Multicellular Systems,” we analyze how multicellular systems organize mobility and communication, by focusing on the role of the vascular system, of mobile immune cells, and the nervous system. In Section “Concluding Remarks,” we conclude with a recapitulation and a discussion of the implications of this theoretical framework for our understanding of distinctively multicellular phenomena such as cancer and aging.

## Why Multicellular Systems are Not Just Balls of Cells: The Limits of Current Accounts of Multicellularity

Multicellularity is a highly diversified phenomenon. Having emerged independently over 25 times in the history of life on earth (Grosberg and Strathmann, 2007), it is realized, in different ways and with different degrees of integration, by bacterial biofilms (Shapiro, 1988; Ereshefsky and Pedroso, 2012), and by eukaryotic systems giving rise to social organisms (Strassmann and Queller, 2010), colonies, chimeras, clonal, and aggregative entities (Herron et al., 2013), plants, fungi, and metazoa. The literature on multicellularity is characterized by a proliferation of accounts that aim to capture the distinctive features, and internal differences, of this class of biological organizations in general^1^.

Most accounts of multicellularity are characterized by a specific attention to reproduction and evolution, and aim to provide an understanding of multicellular systems as evolutionary individuals (Buss, 1987; Santelices, 1999; Michod et al., 2006; Michod, 2007; Pepper and Herron, 2008). In doing so, they highlight features such as reproductive bottlenecks, differentiation between reproductive and non-reproductive tasks, high-cooperation and low conflict, as central to account for the capability of multicellular systems to work as “bundles of adaptation”, where all elements work toward a common evolutionary goal (Queller and Strassmann, 2009; Strassmann and Queller, 2010; Herron et al., 2013).

Yet this is not the only way to look at the problem. While not denying the importance and role of evolutionary considerations for the study of the origins and the histories of the lineages of multicellular systems, another possible research avenue is to investigate the distinctive features of their physiologies. This alternative approach implies looking at how these systems are organized and how their organization is necessary for their persistence. In this paper, we pursue this latter strategy and we focus on the capability of multicellular systems to realize viable dynamic physiological networks capable of self-production and self-maintenance^2^.

In the past, this type of approach has been carried out mostly by taking the living cell as the paradigmatic case (see Moreno and Mossio, 2015). Consequently, organismic and organizational frameworks have been primarily developed by focusing on unicellular life. Multicellularity, however, presents additional challenges to our understanding of biological systems, related to how cells are capable to live together in higher order entities, in such a way that some of their features and behaviors are constrained and controlled by the system they realize. Whereas in the case of cellular systems, this goal has been pursued by focusing on molecular and macromolecular mechanisms and structures, in the case of multicellularity, it needs to be pursued by focusing on *cells and extracellular structures*, by showing how these components are physically and functionally integrated into cohesive systems.

Functional integration is a central concept in order to understand how different types of multicellular entities give rise to viable systems in which the activity of individual cells is recruited and coordinated. In a minimal sense, functional integration consists in the degree in which in a biological dynamic regime of self-maintenance the different components that collectively realize the system as a viable unit depend on one another for their production, maintenance, and activity (see, for example, Bich, 2016).

**Figure 1:**
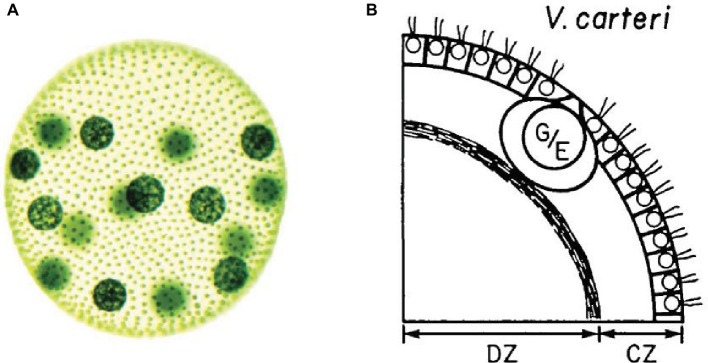
**(A)** Image of an adult *Volvox carteri* (Kirk, 2005, p. 300. *Reproduced with permission from John Wiley & Sons, Inc.*). Gonidia are the bigger spheres; somatic cells are the smaller dots. **(B)** Position of gonidia (G/E) and somatic cells (small circles with flagella) within the *Volvox carteri*’s ECM (Kirk et al., 1986, p. 226. *Reproduced with permission from Company of Biologists Ltd.*).

To achieve functional integration, a biological system requires some internal differentiation – i.e. the presence of components that contribute in different ways to the realization of the system (Mossio et al., 2009) – which constitutes the basic requirements for division of labor. Most evolutionary accounts of multicellularity put special emphasis on the differentiation between germ and soma cells and on their mutual dependence on a reproductive and evolutionary scale (Buss, 1987; Michod et al., 2006). A minimal case of this type of cell differentiation in multicellular systems can be found in one species of the Volvox genus, called *Volvox carteri* (Kirk, 1998, 2005; Herron, 2016; Matt and Umen, 2016). This collective entity is realized by green algae of the order Volvocales, which give rise to a spherical system of thousands of cells immersed in a highly differentiated, modular ECM. As a result the system is capable to move as a unit in space toward sources of light to perform photosynthesis (Figure 1).

*Volvox carteri* is characterized by the differentiation into only two types of cells: somatic and germ cells. Both types of cells are capable of photosynthesis. Somatic cells are equipped with flagella and located at the periphery of the system. They are immersed, with a fixed orientation, in a complex ECM structure differentiated in zones which constitutes most of the mass of the whole system. Due to their specific positioning, these cells provide a coherent propulsion for the whole system. Germ cells are bigger in size and are located inside the system.

The integration between the two types of cells accounts for the maintenance of this class of systems on the inter-generational and phylogenetic scales. Yet, in a system such as *Volvox carteri*, the functioning and maintenance of the system as a whole at the *physiological scale* is not achieved through cell differentiation, insofar as the germ cells do not play a specific role in it. Focusing on differentiation into somatic and germ cells does not say much about how the current system is maintained physically and functionally cohesive and, ultimately, alive during its ontogenetic time scale.

To understand how the current system, rather than the lineage, is maintained, one has to focus on its physiology and, in particular, on the fundamental metabolic functions and mechanisms of intercellular control and communication. When considering the problem from this perspective, several types of features have been proposed as necessary for multicellularity, including genetic homogeneity (Santelices, 1999) and unicellular bottlenecks (Grosberg and Strathmann, 2007), low conflict (Queller and Strassmann, 2009), metabolic integration (Pfeiffer and Bonhoeffer, 2003), genetic control (Arnone and Davidson, 1997; Sebé-Pedrós et al., 2017), patterns of self-organization (Newman and Forgacs, 2005), etc. In particular, two closely interdependent characteristics have been suggested as distinctive of multicellularity and crucial for the functioning, maintenance, and viability of multicellular systems: cellular differentiation (Bonner, 2000; Wolpert and Szathmáry, 2002; Arnellos et al., 2013; Veloso, 2017) and increase in size with respect to unicellular systems (Bonner, 2000; Knoll and Bambach, 2000; Rueffler et al., 2012).

**Figure 2:**
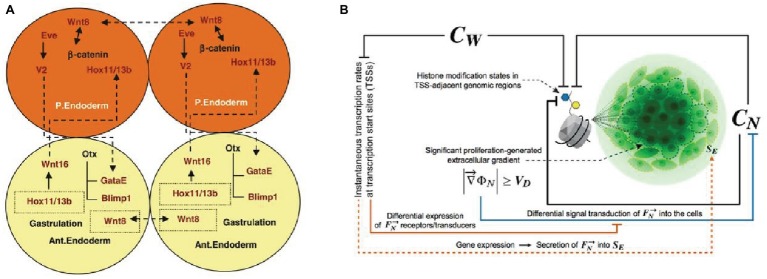
Accounts of multicellularity centered on cell differentiation achieved through intercellular signaling in the development of metazoa. **(A)** The intercellular cross-effects of *Wn8* and *Delta* activate *Hox1/13b* and trigger cells differentiation (Arnellos et al., 2013, p. 869*. Reproduced with permission from Springer Nature*). **(B)** The role of intercellular constraints in histone modification and consequent cell differentiation and proliferation, with generation of intercellular gradients [Veloso, 2017, p. 90. *Reproduced under the terms of the Creative Commons Attribution License (CC BY)*].

Cellular differentiation is a distinctively multicellular feature. It might seem trivial to say, but unicellular systems can only produce different phenotypes and play distinct functions *in time*. Multicellular systems, from biofilms to metazoa, can instead exhibit several differentiated phenotypes at the same time. Such a capability is an essential requirement for functional integration. Through cell differentiation, multicellular systems become in principle capable to harbor components playing different functional tasks, and hence to realize division of labor under certain conditions. A cohesive functional integration between these different tasks is achieved when the differentiation of functions is coordinated at the system level, and the differentiated components contribute through their activity to the maintenance of the system. By showing the fundamental role it plays in the developmental processes of metazoans (Figure 2), Arnellos et al. (2013) and Veloso (2017) have argued that cell differentiation, through intercellular signaling and the formation of self-organized gradients, is the crucial element to account for multicellularity.

Increase in size has also been invoked by many as a crucial factor in the origin and evolution of multicellular systems (see Grosberg and Strathmann, 2007): a mean to avoid unicellular predators and, in turn, to expand feeding opportunities by predating upon unicellular organisms. From a physiological standpoint, the increase in size achieved through multicellularity allows increased storage reserves, the generation of an internal environment and new metabolic capabilities. Importantly, the increase in size allows to reach the critical mass necessary for differentiated somatic cells to realize division of labor. Following this line of argument, Bonner (Bonner, 1998, 2000) argues that the increase in size comes first (for example in *Volvox carteri*) both logically and historically in the origins of multicellularity, followed by the emergence of intercellular mechanisms of cell differentiation and communication.

From a physiological standpoint, accounts of multicellularity built upon either of these two factors, or both together, exhibit several conceptual limits. Let us start with (intercellularly induced) cell differentiation. This property is not sufficient, or maybe not even necessary, in order to account for functionally integrated multicellularity. Differentiation might not be *necessary* for minimal multicellularity, at least in principle, because basic functional integration would not require different types of cells, but just different types of components, to play different physiological functions that contribute to the activity and maintenance of the system. As we will argue in the next sections, these components can be cells but also non-cellular structures like ECM. Thus, in principle, there can be a functionally integrated system – like *Volvox carteri* – with only one type of somatic cells interacting with ECM structures.

Cell differentiation is not *sufficient* for multicellularity either. Differentiation between somatic and germ cells for example, does not entail physiological functional integration. What would be needed to achieve it by means of cell differentiation, instead, is different types of somatic cells. Moreover, even when somatic differentiation is achieved, it needs to be employed to realize division of labor and integration. And the expression of the functional potential of differentiated types of cells in the maintenance of the system requires that the system has reached a critical size, thus enabling the coordinated activity of a critical number of cells of different cell types.

Finally, cell differentiation alone cannot make sense of the differences between distinct types of multicellular systems. While it might allow distinguishing *Volvox carteri* from other systems, it might fail in other cases. Single species biofilms exhibit at least nine types of cells that are the result of differentiation induced by intercellular signals, like in the case of *Bacillus subtilis* (Lopez et al., 2009; Mielich-Süss and Lopez, 2015), let alone multispecies biofilms. This is comparable to cell differentiation in hydra, one of the simplest metazoan, which has 20 cell types.

Increase in size, while it constitutes an enabling condition for division of labor in systems with cell differentiation, incurs into several problems and unpassable bottlenecks as well. Even in the minimal case of biofilm, the required increase in size cannot be achieved without solving some problems related to the circulation of nutrients in all areas of the system and the elimination of waste and toxic compounds. It also requires the implementation of medium- and long-range mechanisms of coordination of cell fate and behavior and of communication (transmission of signals), that go beyond direct cell-to-cell interaction and self-organized cellular gradients. Diffusion, as the mean to provide the molecules necessary for the functioning of an organized system, puts strict limits to the size of a system. As it has been argued (Knoll, 2011), in order to increase in size beyond a thin layer of cells, all multicellular systems require solving the problem of diffusion by realizing, among other things, differentiated structures for the transport of oxygen, nutrients, and molecular signals.

In sum, focusing either on the increase in size or on signal-induced cell differentiation, or even on both factors together, cannot explain why multicellular systems are not limited to just small balls or thin layers of cells, but instead give rise to complex, differentiated and integrated structures. In our view, something more fundamental is missing to understand the reason why the size and number of cells can increase in such a way to take advantage of cell differentiation and allow cells to coordinate and actually carry out activities with different functional roles. Filling this gap, by developing a framework capable to overcome the limits put into evidence in the current accounts of multicellularity, will be the aim of the rest of the paper.

## The Functional Organization of Space

**Figure 3:**
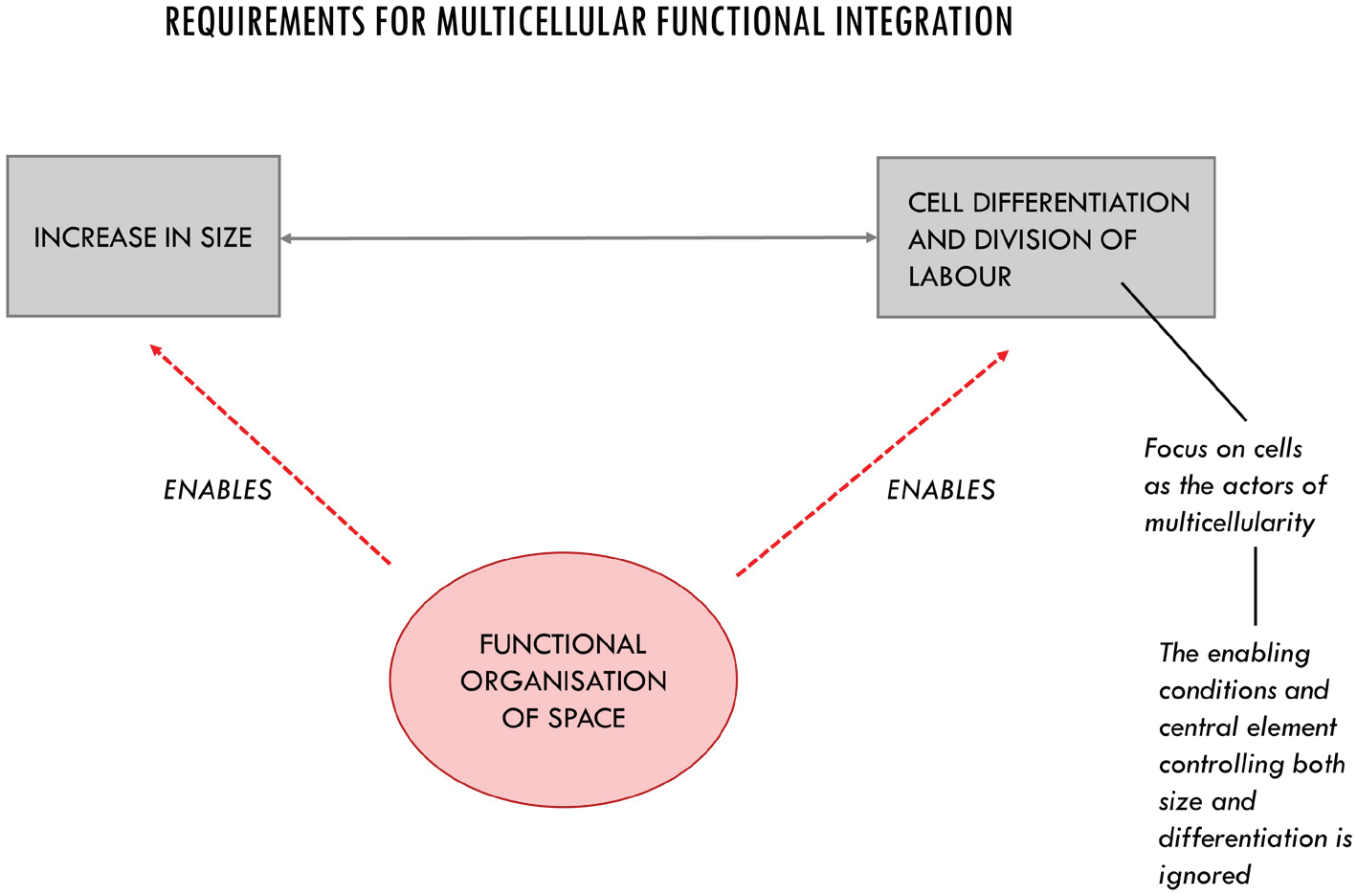
The three main requirements for multicellular functional integration. The organization of space is an enabling condition for the increase in size and cell differentiation. Its functional role in multicellular systems is often underestimated due to an implicit cellular bias.

The missing piece of the puzzle discussed in the previous section, we argue, is constituted by the consideration of the intercellular space: a highly dynamic and differentiated context whose internal organization is of paramount importance for the realization and maintenance of viable multicellular systems. As we argue in this section, the intercellular space is more than just a medium for the activity of cells and for passive diffusion and the storage of molecules. The way it is organized plays a fundamental role in making growth in size and functional differentiation possible (Figure 3).

Increase in size cannot happen without organizing the intercellular space, as it would encounter impassable bottlenecks such as the one constituted by the case of vascularization vs. diffusion. Moreover, dynamic structures of the intercellular space, such as those made of ECM^3^, play a direct role in determining cell fate and behavior and in achieving functional differentiation. They do so by means of mechanical and molecular mechanisms resulting from the 3D anchorage of cells. A fundamental role in this context is played, among others, by mechanotransduction mechanisms responsible for spatial control and proliferation (Alenghat and Ingber, 2002; Wang et al., 2009; Dupont et al., 2011; Piccolo et al., 2014). Variations in mechanical constraints, such as changes of ECM stiffness or changes in cell shape controlled by the ECM, have a profound impact on cell behavior. As showed by Piccolo et al. (2014), for example, transcriptional regulators YAP and TAZ are intracellular mechanisms by which the mechanical properties of the ECM and the cell geometry within the intercellular space instruct cell behavior. YAP and TAZ have been linked to a universal system that controls proliferation, space localization, and organ size. Changes in ECM stiffness and, consequently, in cell shape, constrain the structure of the cytoskeleton and modulate the activity of the YAP/TAZ regulatory transcriptional mechanism: they keep this protein complex in a transcriptionally active state in the nucleus (stiff ECM), or located in the cytoplasm where it is subject to degradation (softer ECM). In other cases, the ECM modulates the activity of cells by acting upon membrane proteins, such as integrins which, in turn, trigger regulatory changes within cells themselves (Adams and Watt, 1993; Hynes, 2009; Rozario and DeSimone, 2010; Tsang et al., 2010; Faulk et al., 2014).

These dynamic structures functionally specify the intercellular space. Despite the increasing amount of data on the involvement of ECM structures in several phenomena of relevance for biology and medicine, their role as active players in the *control*, *coordination*, and *integration* of the components of multicellular systems, together with cells, has been underestimated in the main theoretical accounts of multicellularity and requires further conceptual scrutiny. Supported by the data available, in this section, we proceed in this direction by developing a theoretical framework that takes into account the organization of the intercellular space to the core, starting by addressing the problem of control in multicellular systems. We show that cells are not the only actors of multicellularity. And we argue that the intercellular space, rather than a mere background for cell-to-cell interactions, should be understood as an organized *milieu* populated by extracellular components that play a crucial role together with cells in controlling the dynamics of the multicellular systems.

### The Problem of Control in Multicellular Systems

Control is generally understood in biology as the capability to actively modify the dynamics of a system toward certain states (Rosen, 1970). In living systems, such capability to steer or harness a process, or to modulate the activity of the constituents of the system, can be understood in terms of *constraints*: structures that act as local boundary conditions that enable specific processes and activities. A general definition of constraint can be given in the following terms: given a particular process P, a structure C acts as a constraint upon P if: (1) at a time-scale characteristic of P, C is locally unaffected by P; (2) at this time-scale C exerts a causal role on P, i.e., there is some observable difference between free P, and P under the influence of C (Mossio et al., 2013, p. 164)^4^.

In a nutshell, a constraint reduces the degrees of freedom of processes or collections of elements, in such a way that they exhibit specific behaviors and they can be used to perform some coherent activity in the context of the system (Pattee, 1972, see also Kauffman, 2000; Umerez and Mossio, 2013; Winning and Bechtel, 2018). A biological example of a constraint is an enzyme, which by lowering the activation energy necessary for a reaction, catalyzes it toward an otherwise improbable product. At a different scale, another paradigmatic case is the vascular system, which canalizes the stream of blood toward different parts of the organisms, while in unconstrained conditions the process would take place in a very different way and rate by diffusion, and with different outcomes. The peculiarity of living systems with respect to other natural and artificial systems is that they produce some of the constraints – such as enzymes, membranes, or the vascular system – that are necessary for their own internal functioning (Montévil and Mossio, 2015; Moreno and Mossio, 2015).

Distinct types of constraints play different functional roles. A crucial distinction can be made between those *structural constraints* which statically and passively reduce the degrees of freedom of the processes they canalize, and those dynamic *control constraints* that actively select between the degrees of freedom available (Pattee, 1972). Structural constraints can be realized by static structures, or by purely physical interactions like in the case of the restriction of spatial freedom by neighboring cells. Control, instead, as the capability to actively modify the dynamics of a system toward certain states, requires the presence of dynamic constraints that are characterized by both sensory and effector capabilities, and that exhibit differential activity (e.g., activation or inhibition) in presence of specific boundary conditions or interactions, for example, with signal molecules (Bich, 2018). Control constraints do not reduce degrees of freedom once and for all. Instead, they modulate the controlled processes depending on their activation status^5^.

Control is crucial for an organized system, such as a living one, in which processes are stochastic, and constantly require modulation in relation to changes in external and internal conditions. Moreover, different subsystems might present different ways of operating, and their activities and rates need to be coordinated to avoid conflict and to ensure their joint functional contribution to the maintenance of the system. To do so, the activity of basic control constraints is directly modulated by other specialized regulatory constraints in the system. The result is the realization of control architectures capable to implement those of differential and specific responses necessary for the integration and coordination of the activities of several subsystems and, ultimately, the maintenance of a complex organization (Bich et al., 2016).

Addressing the question of functional integration in multicellularity requires taking into consideration how control is realized at the intercellular scale. At this level, what is controlled and coordinated is, among other things, the activity of cells. Cells are themselves living autonomous entities that exhibit agential capabilities, and which need to be organized into integrated and cohesive systems, by avoiding conflict and making their activities compatible and mutually sustaining (Soto and Sonnenschein, 2018).

Focusing on the specificity of control at the intercellular level requires considering how a system is capable to constrain and coordinate the activity of cells together with that of other extracellular components at short, medium, and long ranges, in such a way that they achieve a viable way of living together. For example, if we think of proliferation and motility as the properties that are characteristic of unicellular organisms – their “default state,” as defined by Soto and Sonnenschein (2011) – a challenge multicellular systems face is how to exert a differential and dynamic control upon these properties in a way that is functional for the whole. Not all the cells can proliferate and not at any time. Therefore, the system activates the division of certain cells in specific moments in time and inhibits it in others. Moreover, depending on the state of the system, the capability of *motility* is also inhibited in most cells. When those constraints that act on proliferation, motility, mobility, etc. fail, or their properties are modified, these changes may give rise to different forms of multicellular organization, more often incompatible with the original one, such as in cancer, and contribute to the development of several human diseases, such as osteoarthritis, fibrosis, etc. (Bonnans et al., 2014).

### Spatial Control and the Organization of the Intercellular Space: Overcoming the Cell Bias

In order to understand multicellular control, it is important to put into evidence three closely interconnected theoretical aspects. The first is that control is not exhaustively accounted for by cell-to-cell interactions alone: extracellular components produced by the system also play an active role in controlling the dynamics of the system and contribute to its realization and viability. Second, these additional control subsystems populate and functionally specify the intercellular space, which is not an inert background for cells, but a dynamic *milieu* which constitutes the boundary conditions for cells activity and whose properties directly influence the behavior of cells. Third, as a result, this space is functionally organized in such a way that it enables increase in size and division of labor at the system’s level.

One of the reasons why the full role of the organization of the intercellular space and of extracellular control structures has been overlooked as a theoretical principle in understanding multicellularity – for example, in favor of cell differentiation based on cell-to-cell interactions – lies in an implicit cellular bias, which identifies in cells the *only* active players in this class of biological systems. Placing the focus on cells is not surprising, and it is also correct, albeit partial. Cells are the most fundamental biological units. As correctly stated by Pier Luigi Luisi, a biochemist and synthetic biologist involved in origins of life research “It is well known that life is cellular and only cellular: all tissues and organs of all animals and plants are organized assemblies of cells – so that we can consider the cell as the elemental constituent of life on this planet” (Luisi, 2017, p. 353). Whereas this statement may be coherently and successfully applied in research on origins of life – i.e. how the first living cells originated – it would be problematic if, when addressing multicellularity, the statement “all life is cellular” was interpreted as the fact that cells are all that is important, or the only building blocks necessary to understand multicellular systems.

Even accounts of multicellularity which take space into account, such as Wolpert’s concept of “positional information” (Wolpert, 1969), do not address the properties and organization of the intercellular space *as such*. Instead, they assume a perspective centered on cells, and focus on: (1) how cells detect their surroundings, interpret their position in space, and change their activity accordingly and (2) how spatial differentiation results from a “process by which the individual cells within a population are specified to undergo a particular molecular differentiation, which results in a characteristic spatial pattern” (Wolpert, 1969, p. 2). More recent accounts also address space from a cellular perspective, by putting into evidence the importance of the transition from a temporal differentiation to a spatial segregation of cell types in the origin of multicellularity (Brunet and King, 2017)^6^.

Yet “all life is cellular” does not necessarily mean that cells (or groups of cells) are the only active functional components in multicellular systems. In fact, there are other dynamic components produced within a multicellular system, such as ECM structures, that play an active role in it and provide a decisive functional contribution to its maintenance and integration. They do so by acting through spatial relations. It is specifically ECM structures that exert a fundamental constraining function upon the cellular default state of proliferation and motility in multicellular systems (see, for example, Montévil et al., 2016). The ECM gives rise to structures that exhibit a trade-off between stability and dynamicity which allows them to play a control function within multicellular systems. These extracellular components cannot be understood only as *structural constraints* which statically reduce the degrees of freedom of the constrained cells once and for all. They are dynamical components that actively select between the degrees of freedom available, thus exhibiting – together with cells – the basic sensory-effector capability characteristic of *control constraints* at the intercellular level.

While providing stable anchorage, and exhibiting specific features in different tissues, ECM structures also carry out differential constraining activity that functionally modulates the state of cells. They are dynamical constraints because, at different physiological time-scales, they can change their physical state, density, composition, 3D shape, or the state of activation of their proteins, in relation to the state of the system or of a specific tissue. For example, mechanical forces and molecular interactions can alter the functional domains of proteins embedded in the matrix; building and dissolving the matrix also selectively modifies its control capabilities in time. In addition, enzymes can act as regulatory switches that modulate the control capabilities of the ECM by creating and modifying collagen cross-links (Chen et al., 2015).

**Figure 4:**
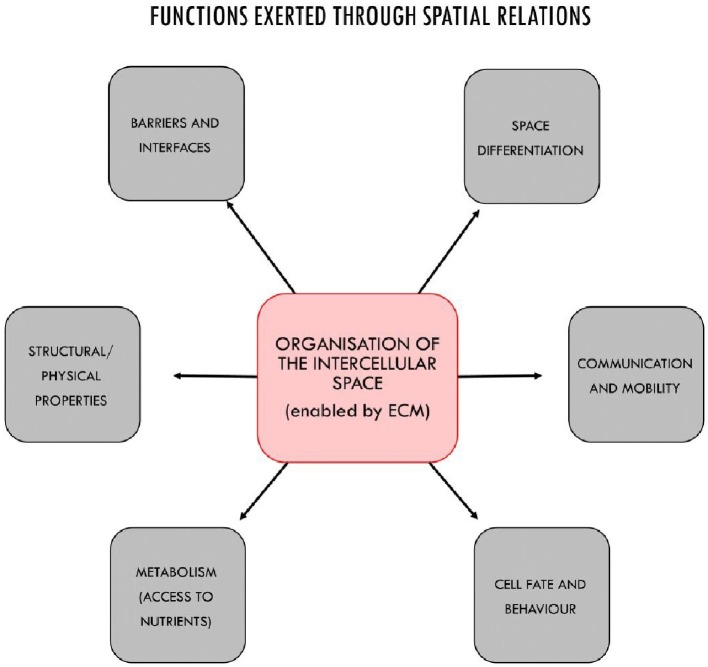
The main functional properties of multicellular systems realized through the organization of the intercellular space, in most cases by ECM structures acting as control mechanisms together with cells. The functional features of the intercellular space include: the control of cell fate and behavior; the enablement of metabolic capabilities by providing access to nutrients (e.g., through vascularization); physical properties such as resilience to physical stress and structural cohesiveness; the constitution of basement membrane for anchoring epithelial or endothelial cells, tendons, bones, etc.; spatial differentiation and modularity with distinct areas characterized by different boundary conditions for cells, and the realization of specialized areas and tissues; the creation of permeable or semipermeable barriers and interfaces by contributing to structure the epithelium, or directly, like in the kidney; and finally, the organization of mobility and communication at medium and long range (beyond cell-to-cell signaling).

In turn, depending on their (activation) state, ECM structures can constrain in different ways the behavior of cells, by acting upon specific membrane receptors, by inducing changes in cells shapes, or by modulating the activity of signaling molecules and morphogens. These activities are functional insofar as they contribute to the overall maintenance of the system (Figure 4). The ECM, for example, modulates cell fate and activity by interacting with mechanosensitive proteins such as integrins in cell membranes and by activating gene transcription (Rozario and DeSimone, 2010; Dupont et al., 2011; Piccolo et al., 2014). As dynamic repositories of signal molecules, morphogenetic and growth factors, ECM structures are capable of modulating their availability to cells on the basis of specific interactions in the intercellular space, thus controlling the behavior of cells both at short and medium ranges (Hynes, 2009; Rozario and DeSimone, 2010; Tsang et al., 2010). Changes in the stiffness of the matrix also control cell differentiation (Lv et al., 2015) as well as migration, apoptosis, and proliferation (Wells, 2008). These features are not limited to eukaryotic multicellular systems; the matrix plays similar roles in bacterial biofilms as well (Sutherland, 2001; Branda et al., 2005; Steinberg and Kolodkin-Gal, 2015).

In metazoa, ECM structures interact with cells in assembling some of the supra-cellular structures that give rise to space differentiations and play a functional role as boundaries and interfaces: i.e. the endothelium with respect to the vascular space, and the epithelia in the case of tissues. Epithelial tissues play a fundamental role in animal development and in the organization of complex animal bodies (Tyler, 2003; Arnellos and Moreno, 2016). They also support animal movement and sensory-motor capabilities (Keijzer and Arnellos, 2017). The formation of the epithelium is not determined by the intrinsic properties of cells only. The ECM generally plays an active role in controlling the features and positions of cell-cell junctions and, consequently, cell assembly (Tseng et al., 2012). In the specific case of the epithelium, the ECM structure involved in the assembly and stabilization of this organized intercellular structure is the basement membrane (BM). Alteration in the properties of the BM in relation to the epithelial cells – through release of cell-BM contacts or through degradation of the BM by proteolytic enzymes – is associated with and can trigger the epithelial-mesenchymal transition (EMT), with strictly controlled or chaotic disaggregation of the epithelial organization. These processes are involved in the formation of tissues and organs during development and in cancer, respectively (Nisticò et al., 2012).

All together, the functions exerted within an organized intercellular space make fundamental contributions to the realization of the two features that are usually considered as the basis of multicellularity: increase in size on one side and cell differentiation with division of labor on the other. The former is enabled, among other things, by the control exerted by the ECM upon cell proliferation, and by the fact of providing structural support and cohesiveness to the system, anchoring for cells, and acting as a scaffold for shape transitions during development (Sherwood, 2015)^7^. Moreover, increase in size is also achieved by means of spatial organization that makes it possible the distribution of nutrients to cells, for example through vascularization.

The ECM also controls cell differentiation and contributes to coordinate the activity of different cell types in such a way that the system achieves division of labor and functional integration. Matrix structures modulate cell differentiation by sensing and transducing mechanical signals that have precedence over short range cell-to-cell control mechanisms (Guilak et al., 2009; Piccolo et al., 2014; Lv et al., 2015, see also Streuli et al., 1991). By controlling increase in size, they allow the system to achieve the critical mass that is necessary to take advantage of the presence of different types of cells. By exerting control at medium ranges, they can coordinate the activities of groups of cells and specify tissue phenotype (Lelièvre et al., 1998). In particular, matrix structures exhibit properties specifically related to their localization in the system. ECM’s exhibits specific features vary from tissue to tissue, thus exerting a distinctive type of control upon cells in each one. In such a way, it provides a decisive contribution to the spatial localization, differentiation, and stabilization of specific cell types in distinct tissues, and it contributes to functionally employ the potentiality provided by the spatially concentrated, coordinated activity of different cell types to realize organ differentiation and division of labor^8^.

In sum, the intercellular space cannot be considered a mere background for cells. Its functional organization – especially, due to the control capabilities of ECM structures – on the one hand accounts for the possibility of increasing the numbers of cells living together. On the other hand, it puts together, integrates, and coordinates the activity of several types of cells toward physiological goals. In such a way, it enables division of labor by allowing types of cells to realize different functions and give rise to tissues and organs: the latter considered as integrated ensembles of cells capable to perform functions, in the physiology of an organism, that are necessary to maintain this organism alive.

## Motility, Mobility, and Communication Within Multicellular Systems

When a multicellular biological system grows in size and in the number and types of cells, it becomes more and more necessary to coordinate the activity of different components at medium and long ranges and to transport nutrients everywhere in the system. Multicellularity has solved this problem of spatial organization in different ways and degrees, by developing internal control and communication mechanisms that coordinate the parts of the systems at distance.

The solution to the issue of integration at longer spatial scales is achieved by organizing movement: i.e., by controlling what moves in space, how it does so, and when. Movement, which is a widely shared property in unicellular organisms, needs to be under control to realize multicellularity. Unlike in unicellular organisms, motility is inhibited in most cells of multicellular systems. This is true for all multicellular systems, including biofilms. Inhibiting motility prevents the disaggregation of the system, and it allows to spatially concentrate groups of cells that collaborate to perform certain functions^9^.

The functional properties involved in the organization of the intercellular space play a primary role in the control of motility. In bacterial biofilms, motility is directly modulated by the matrix, which mechanically inhibits the rotation of the flagella and triggers intracellular signal cascades^10^. As a result, the immobile bacterial cells differentiate into *persisters*, which increase the production and deposition of matrix molecules and, in turn, inhibit the motility of other cells, thus further amplifying matrix production with a cascade effect that contributes to the overall growth of the biofilm (Cairns et al., 2014; Steinberg and Kolodkin-Gal, 2015; Waters et al., 2016). Moreover, the inhibition of motility contributes to the realization of a regime of spatial and functional differentiation, where groups of cells or mixed-species microconsortia locally share a similar extracellular environment and work together, thus contributing to division of labor (Flemming and Wingender, 2010).

In *Volvox carteri*, the ECM controls the motility of flagellated somatic cells. By immobilizing them in place on the exterior of the system with a fixed outward orientation, the ECM prevents them from swimming independently, dispersing and, thus, disaggregating the system. At the same time, it enables them to perform their function, which consists in collectively realizing a coherent movement for the whole system (Kirk, 1998).

In metazoa, the ECM also contributes to modulate motility. During specific stages in development, entire groups of cells migrate, and their movements are highly canalized by the dynamic properties of ECM structures. Interaction between ECM and integrins controls cell adhesion and deadhesion. Contractions of the cytoskeleton generate traction on the ECM and allow locomotion in combination with ECM-associated growth factors, signals, cytokines, mechanotransduction, etc. (Rozario and DeSimone, 2010). Moreover, different degrees of stiffness of the ECM allow or inhibit cell motility. Stiffness is influenced by crosslinking of matrix components such as collagen, or by the modification of proteoglycans, for example, by the amount of hyaluronans which connect proteoglycans to collagen. In different tissues and at different times, cells are controlled by the degree of stiffness of the ECM. Experiments show that normal mammary epithelial cells, when growing in a soft 3D matrix similar to the basement membrane found in *in vivo* tissues, adopt an acinar structure organized in spherical monolayers of cells with a central lumen. Increase in ECM stiffness first causes loss in the spherical monolayer organization to give rise to a tight ball of the same cells with no internal lumen and, if further increased, it triggers migratory behavior within the 3D matrix (Halder et al., 2012).

**Figure 5:**
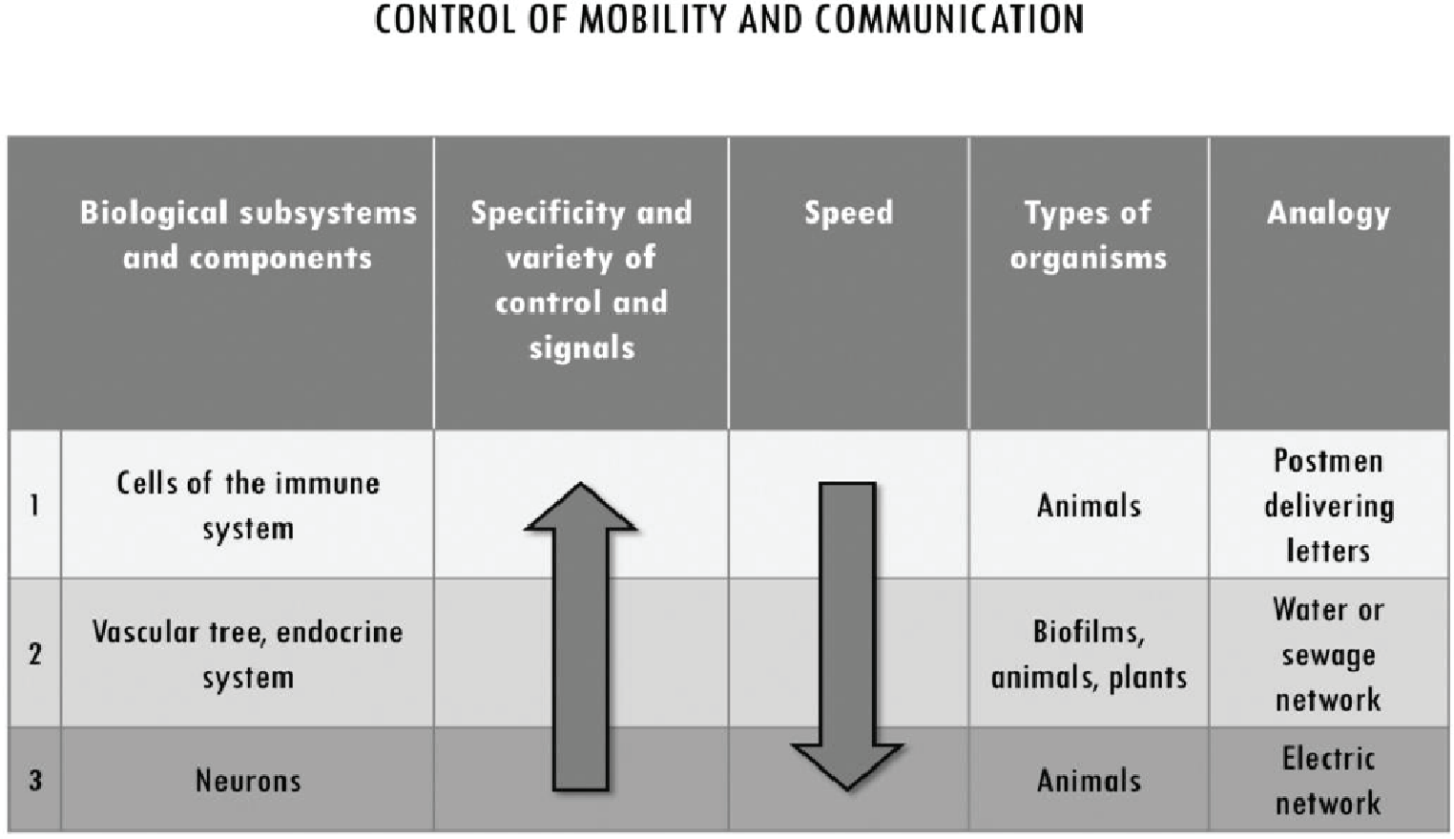
Subsystems for the control of mobility and communication in multicellular systems. They differ with respect to the degree of specificity and the variety of control mechanisms they implement, and for their speed. While vascularization is common to almost all multicellularity, the other subsystems are specific of metazoa. We associated to each of them an analogy from city organization to exemplify their distinctive functioning in multicellular systems. Immune cells’ high specificity and low speed of movement is analogous to a postman delivering letters. The transport of water, nutrients and waste through vascularization can be associated to a hydraulic network which, at least in very basic form, can be found in most human settlements over a certain size. Finally, neuronal architectures in the body can be associated with electric networks in terms of high speed of movement and lower specificity of individual signals.

Given that motility is highly controlled, and most cells are immobile in multicellular systems, the latter employ different ways of delivering signals, of exerting coordinated and localized control when and where needed. As already discussed, ECM structures can exert medium-range control at the tissue level beyond the range of cell-to-cell interactions. In addition to that, multicellular systems achieve integration by organizing space at longer ranges, by controlling the movement of some cells and of those nutrients, signals, control molecules, etc. that are necessary for the coordinated activity of the components in different areas of the system. Long-range control upon movement and communication within the system is achieved in at least three different ways (Figure 5): (1) by making components mobile in a fluid through vascularization; (2) by means of cells, such as the immune ones, that retain the capability of motility and move in the blood or through the ECM in tissues; and (3) through signal transmission architectures realized by networks of neurons.

When the size of a multicellular system increases, and the cells in the interior do not have direct access to the external medium, directly or through diffusion, mechanisms that distribute nutrients to all the cells are employed. Vascularization plays a fundamental role in this respect, by providing all cells with an efficient access to nutrients, oxygen, and other essential molecules. It constitutes the fundamental long-range space-organizing subsystem in multicellularity, specialized in the control of movement of nutrients, hormones, signal molecules, and cells, by making them mobile within the flow of a liquid medium. Vascularization is common to all multicellular systems which exert control upon their internal mobility. This is also true for biofilms, where the organization of the hydrophobic molecules of the matrix gives rise to channels that harness the flow of fluid and allow nutrients from the periphery to reach the bacteria residing in the inner region (Sutherland, 2001; Cairns et al., 2014). It is absent from few multicellular systems that, like *Volvox carteri*, do not have integrated metabolism and have their somatic cells localized on the outside.

The ECM contributes to the development of vascularization and to the organization of the vascular tree. It constitutes one of the control mechanisms for the differentiation and for the modulation of the behavior of endothelial cells (Newman et al., 2011). More specifically, the ECM basement membrane of vessels (VBM) provides growth factors, signals and the mechanical support for the construction of blood, lymphatic, and kidney tubule vessel structures. Growth factors such as VEGF-A (vascular endothelial growth factor A) are stored in the VBM and can be released to stimulate the differentiation and proliferation of cells in vasculogenesis and angiogenesis (van Genderen et al., 2018).

The strict relationship between vascularization and growth of the system can be observed in tumors. In humans, tumor may have limited growth in 3D (1–2 mm^3^) before their demands of oxygen and nutrients cannot be met by diffusion alone. When cells become hypoxic, to enable further growth a tumor promotes several distinct mechanisms of vascularization to properly bring oxygen: e.g., sprouting and intussusceptive angiogenesis, mobilization of endothelial progenitor cells from the blood, and vasculogenic mimicry by tumor cells that differentiate to an endothelial phenotype and realize tube-like structures. These processes of neovascularization are carried out through the activation of endothelial cells by means of growth factors from the FGF (fibroblast growth factors) and VEGF families stored in the matrix, and through the remodeling or the co-opting of ECM structures (Hillen and Griffioen, 2007).

While vascularization is a common feature in multicellularity, other mechanisms are more specific of animal life. In metazoa, not all cells lose motility or rely only on vascularization for their transport within the system. The immune system is a way to organize movement and communication by controlling, in a way that is functional for the whole, those cells that have retained intrinsic motility.

Immune cells can move through blood, but in most cases they reside in tissues, where they are highly mobile within the ECM network that fills the space between tissues cells (Purwar et al., 2011)^11^. There, these primed and memory cells, called “T resident memory cells”, provide for a primary system of immune surveillance at the level of tissues and organism’s barriers. Through their mobility among the cells that constitute the tissue, they can exert a localized and specific control. By delivering highly specific signals to cells within tissues, they play important fine-grained coordinating functions, such as, among others, tissue repair, the regulation of fat cell metabolism to adapt to prolonged exposure to environmental cold (Lee et al., 2015), and communication with the nervous system in the guts (Gabanyi et al., 2016). In the case of zebra fish, they even connect xanthoblasts to melanophore, which then can be loaded with melanin and give rise to the stripes that characterize this fish (Eom and Parichy, 2017). The movement of immune cells in tissues is afforded by the porosity of the molecular network that makes up different types of ECM, depending on the orientation and density of the fibers. It is made possible also by the ability of immune cells to modify their shape, which in turn is limited by the nuclear size and shape and by its intrinsic ability to deform as well. Changes in the properties of the ECM, such as its porosity, can modulate this mobility of immune cells in the extracellular environments.

The third subsystem that contributes to the long-range spatial and functional integration of multicellular systems is the nervous one. Specific of metazoa, it establishes quick long-range communication networks that enable the control of the activities of a large number of cells at short time scales, thus allowing fast coordinated behavior. This is consistent with the thesis on the origin of neurons advanced by Keijzer et al. (2013) as control mechanisms for the activity of muscles: the function for which neurons first evolved in metazoan was to enable muscles to coordinate their contractions by transmitting signals between muscles and realize a synchronized propulsion movement, like it happens in the jellyfish^12^.

## Concluding Remarks

In this paper, we addressed the problem of multicellularity from a physiological point of view, focused on how the components of a multicellular system are functionally integrated into a viable organization. We argued that the limitations of the main accounts of multicellularity, based on increase in size and cellular differentiation to explain this phenomenon, depend on an implicit cell bias – that sees only in the cells the active players within multicellular system – and are missing an important conceptual point, that is, the organization of the intercellular space and the role of extracellular structures in it. Realizing a multicellular system, instead, requires solving the problem of controlling, integrating, and coordinating the activity of the components at different spatial scales and providing the nutrients and oxygen necessary for their maintenance.

We provided a theoretical framework to understand the role of spatial organization in multicellular systems, based on the role (1) of ECM structures as control mechanisms that organize the system at short (together with cell-to-cell interactions) and medium ranges and (2) of vascularization, immune cells, and neural cells, which control movement and communication at longer ranges. The central idea is that the intercellular space is internally differentiated and functionally organized by these dynamic extracellular (ECM) or supracellular (endothelium, epithelium with their BMs) structures that play an active role as control mechanisms. The intercellular space can be understood as functionally organized because it brings together this set of mutually dependent, yet functionally differentiated, extracellular control components that contribute to the viable integration of the system. These components are sensitive to changes in spatial properties, and by acting as selective constraints upon dynamic spatial relations, they control the system’s processes (e.g., transport of metabolites through vascularization), the activity of other cellular or non-cellular components, and the boundary conditions that allow cells to survive and carry out their activity. They are functional insofar as their activities contribute to the overall maintenance of the system.

This approach can provide a theoretical perspective to integrate contributions from the growing field of studies on the ECM and its functions, and to support further research hypotheses. A theoretical framework centered on the organization of space can contribute to provide an understanding of why ECM is so important and why it is crucially involved in all these phenomena. It can provide insights into the main causal factors underlying the distinctive features of different types of multicellular organizations (from biofilms, to plants and metazoa), understood as different ways to organize space and, possibly, of their dependence on differences between types of ECM. It allows also investigating possible parallelisms between the evolution of metazoa and the evolution of ECM molecules such as collagen. As it has been argued in the case of animal multicellularity, many of the capabilities required for it had already evolved in unicellular systems (Grosberg and Strathmann, 2007; Brunet and King, 2017): there does not seem to be massive appearance of radical genetic novelties between metazoa and their closest unicellular relatives. As shown by Sebé-Pedrós et al. (2016), cell differentiation is already present in unicellular organisms such as *Capsaspora*, which has three temporal life stages, one of which is an aggregative multicellular stage with production of ECM. What characterized the origins of metazoan multicellularity might have been the emergence of regulatory novelties related to genetic and spatial control: dynamic regulatory gene networks that allow fine tuning of activities of cells (Sebé-Pedrós et al., 2017), together with the emergence of new ECM molecules and structures such as Collagen IV and basement membrane (Fidler et al., 2017). The genetic requirements for the fine-tuned capability of modulation of cell fate and activity might have proceeded hand in hand with the emergence of new extracellular structures.

Thinking in terms of the organization of the intercellular space has also important implications for our understanding of multicellular phenomena of both biological and medical relevance, such as immunity, cancer, and aging. Until recently, immune cells present in the blood of vertebrates captured most of the attention of immunologists, especially in humans, where blood remains the obvious way to look at the functioning of the immune system. However, it was recently discovered that the amount of lymphocytes in the blood was drastically outnumbered by a factor of 50 by those residing within the tissues and organs (Purwar et al., 2011). These tissue resident memory T lymphocytes are seeded into the tissues from the blood in the course of an immune reaction. Yet they do not recirculate back to the blood, but stay *in situ* within tissues for the entire life of the organism. There, they are very active patrolling these tissues, where they constitute the first line of defense. They can do so because they have the capacity to move between the cells that constitute the tissue, through a space filled with the ECM, as discussed in Section “Motility, Mobility, and Communication Within Multicellular Systems” (see also Ariotti et al., 2012).

The link between immune protection and mobility became obvious when studying certain hereditary immune deficiencies which are uniquely induced by impeding solely the capacity of immune cells to move, such as by knocking down actin remodeling, for example, by deadly mutation of DOCK8 or Coronin 1, two gene products necessary for actin remodeling. Crippling down the motility of these cells creates by its own a deep immune deficiency. Importantly, when observed *in vitro* in a liquid medium, these mutated lymphocytes do not show any defect, whereas, when put in a 3D matrix, they prove to be incapable of movements and die by plasma membrane ripping (Zhang et al., 2014).

Numerous intimate immune functions rely on the ability of the cells mediating them to be mobile. This is the case for T regulatory lymphocytes which need to be mobile to move toward the sites where they carry out their regulatory activity, even though some of them could be generated *in situ*. Another demonstrative example is provided by the way cytotoxic T lymphocytes can get rid of the influenza virus. It has been known for a while from experiments with mice that the survival of the mouse relies on a vigorous response by CD8+ T lymphocytes, which can kill parenchymal infected cells, thus exhausting virus replication. Obviously, as these effector cells are generated outside the infected tissues, they need to move to the place where infected cells are. This condition can only be realized if they could follow cues left within the ECM by neutrophils under the form of tiny fraction of their cytoplasm loaded with chemokines attached to the scaffold of ECM (Lim et al., 2015). Here, again the importance of mobility and its 3D organization within the ECM finds its necessity and proves to be a major explanatory factor.

The organization of the intercellular space plays also an important role in cancer. Cancer could be regarded as an organoid including tumor cells and normal cells such as endothelial cells or fibroblasts, also set around the organization of a heterogeneous scaffold of ECM (Marie and Merlino, 2019). The high stiffness and cross-linking of the ECM that can be found in tumors, provide cancer cells with the necessary signals of proliferation and/or invasion, transmitted through the mechanotransduction pathway of signaling (Halder et al., 2012; Acerbi et al., 2015). Pioneering work in cancer biology has advanced the thesis that alteration of ECM may anticipate cell transformation (Bissell and Radisky, 2001; Soto and Sonnenschein, 2011)^13^. At the same time, and for the same reasons, tumor ECM shields the tumor from the possible invasion of immune cells. This aspect of cancer biology is under scrutiny and may even change the therapeutic way with which we consider acting on tumors, or even on the immune system, in the case of cancer. For example, if the physical properties of ECM are structuring the relationships between itself and the immune system, one can envision ways to externally (physical agents) or internally (enzymes regulating the ECM scaffold) act on it in such a way as to have a deep effect on the tumor itself.

Many other fields in biology and medicine could benefit from thinking in terms of the active organization of space by the ECM (Lampi and Reinhart-King, 2018). The case of aging is of some interest in this regard. Changes in the properties of the ECM are involved in the aging of vascular systems (Kohn et al., 2016). Moreover, as mentioned previously in this section, the intrinsic alteration of lymphocytes motility can lead to a profound state of immune deficiency. During aging, the alteration of the physical properties of the medium where lymphocytes are moving, namely ECM, could similarly lead to severe and progressive alterations of the immune system and its responses. So far, a unifying picture of the mechanisms of aging is not available. Yet, for what concerns the aging of the immune systems (immunosenescence), it is possible to imagine or even find in the literature clues leading to think that the alterations of ECM known to occur during aging and to be an hallmark of it, may severely impinge on cell differentiation and stem cells numbers, thymus aging, T-cell repertoire shrinkage, to cite few, or even on the individual inflammatory status. Their effect is to create a state of immune deficiency similar to the one found in the hereditary diseases mentioned previously, as discussed in Moreau et al. (2017). Increase in ECM stiffness due to progressive crosslinking of matrix proteins associated with a low turnover, profoundly affects the possibility of lymphocytes to travel through it. Changes in ECM porosity affect another important parameter in cell biology, which is cell deformation itself, linked to nuclear size, shape, and deformability, as the nucleus is the biggest organelle of a given cell. Nuclear mechanical stress is a new part of biology which begins to produce new interesting data, shedding light on new mechanisms of DNA mutations, rupture of nuclear membrane, changes of epigenetic traits, to cite a few.

In sum, the importance of a theoretical framework that focuses on multicellular systems in terms of organization of the intercellular space and the relative functional properties, does not only contribute to an understanding of how multicellular systems are organized and to the formulation of research hypotheses on their origins and evolution. It also provides a unified perspective that puts together different work on ECM and on spatial functional features of multicellular systems, to understand failures of multicellular organizations, with important implications for medicine.

## Author Contributions

J-FM provided the original idea. LB and J-FM developed the theoretical framework and wrote Section “Concluding Remarks.” LB wrote Sections “Introduction,” “Why Multicellular Systems Are Not Just Balls of Cells: The Limits of Current Accounts of Multicellularity,” “The Functional Organization of Space,” and “Motility, Mobility, and Communication Within Multicellular Systems” of the manuscript and drew the diagrams. All authors discussed the general outline of the article and contributed to comments and revisions.

### Conflict of Interest Statement

The authors declare that the research was conducted in the absence of any commercial or financial relationships that could be construed as a potential conflict of interest.

## Footnotes

^1^A special interest has been placed upon the subclass of metazoa, because they exhibit the highest structural and behavioral complexity, and the degree of cohesiveness that we usually associate to organismality. See, for example, Arnellos and Moreno (2016) for a detailed review of this literature. We will not focus here specifically on the issue of multicellular organismality but, rather, on the distinctive features of multicellular organizations in general.

^2^This approach is in agreement with the line of investigation in physiology traditionally inaugurated by Claude Bernard, centered on the idea that the activity of the components of a biological system contributes to the *realization* and *stability* of their internal milieu which, in turn, is the condition for their own existence (Bernard, 1865, 1878; see also Bechtel, 2007; Mossio and Bich, 2017).

^3^The eukaryotic ECM is a complex three-dimensional structure that characterizes the intercellular space. It is a network of several types of proteins and carbohydrates (collagen, enzymes, glycoproteins, proteoglycans, etc.) which can include several types of other molecules, such as growth factors (see Hynes, 2009; Mecham, 2011 for an overview). Bacterial biofilms are also characterized by a specific extracellular matrix, which has different composition and structure compared to those found in eukaryotic systems, notably including DNA (see, for example, Steinberg and Kolodkin-Gal, 2015).

^4^A more detailed characterization can be found in Montévil and Mossio (2015).

^5^David Fell, for example, in a classic textbook on metabolism in cellular and multicellular systems, stresses these features of control: “we can regard metabolic control as the power to change the state of metabolism in response to an external signal” (Fell, 1997, p. 3).

^6^Accounts focused on intercellular self-organization and the formation of patterns of cell, from Weiss’ (1968) to Newman’s (Newman and Forgacs, 2005), are characterized by a similar conceptual attitude according to which the distribution in space is understood as resulting from cell-to-cell interactions more than a causal actor in itself.

^7^A minimal example this latter function can be found in *Volvox carteri* where the ECM structures that connect cells play a crucial role in in the development of the system. After cleavage, the embryo cells are in an inside-out configuration that does not allow directional swimming toward light sources: the gonad cells are on the outside and the flagellar ends of somatic cells on the inside. At this stage, the embryo inverts by means of changes in cells shapes and using cytoplasmatic bridges between cells as pivots. It brings the flagella on the exterior of the system and sequesters the gonad cells on the interior (Kirk, 1998, 2005). The role of ECM structures in this process of development is shown by mutants. The extra-embryonic vesicle needs to expand to give enough space to allow inversion, and this does not happen in Inv C and InvB mutants, where the secretion of extra-embryonic ECM is affected, modifying the properties of the vesicle (Matt and Umen, 2016). Other mutations affecting the properties of the ECM cause the system to disaggregate just at the end of inversion, as cells are not kept together and disperse (Kirk, 1998). Moreover, at the end of inversion, the production and organization of the ECM plays also a central role in establishing the orientation of the flagellated cells (Hallmann and Kirk, 2000).

^8^As previously argued (Section “Why Multicellular Systems Are Not Just Balls of Cells: The Limits of Current Accounts of Multicellularity”), cell differentiation is not always found associated with division of labor in a functionally integrated system. In fact, a minimal example of functionally integrated multicellular system is for example *Volvox carteri*, which is characterized by two types of physiologically active components – i.e., the flagellated somatic cells and ECM structures – despite exhibiting only one somatic cell type. The integration between these two types of components allows the system to behave as a unit, capable to swim toward light sources during the day and toward the ground during the night. The ECM constitutes around the 99% of the volume of the system (Adams, 2013). In addition to contributing to development, to giving structural cohesion, and to enabling coordinated movement by organizing the position of the flagellated cells, the ECM plays other physiological roles (Kirk, 1998). ECM performs enzymatic activity by providing the system with an extracellular phosphatase with a broad substrate specificity which is used when reaching the phosphate-rich ground during nights (Hallmann, 1999). The liberation of daughter spheroids occurs through local enzymatic process of lysis of the ECM (Sumper and Hallmann, 1998). ECM promotes gonadal growth by storing nutrients, and gonadal cells grow twice as fast then when ECM is ruptured (Hallmann and Kirk, 2000). ECM is also involved in sexual induction through the phosphorylation of ECM glycloproteins in response to pheromones with cAMP as second messenger (Kirk, 1998; Hallmann, 2003).

^9^Cases in which the multicellular system fails to functionally control motility include for example metastasis.

^10^Changes in the properties of the matrix can reverse the process in distinct phases of biofilm development (O’Toole and Kolter, 1998).

^11^For example, there are roughly 20 billion T cells just in the skin of an adult human, twice the total number of T cells in blood (Purwar et al., 2011).

^12^As argued by Keijzer and Arnellos (2017), a basic nervous system allows a faster and more extensively coordinated control upon a contractile body than, for example, the mechanisms employed by sponges, which are based on contractile cells and chemical signaling among them.

^13^See Bertolaso (2016) for a discussion of different approaches to cancer, including those focused on tissue properties as opposed to cell-centered ones. As an alternative to both positions, Bertolaso proposes a dynamical and relational view of biological organization and cancer, focused on the dialectic between different types of processes and on the role played by context. See Green (2019), instead, for a discussion of tumor progression in hierarchical terms as a case of downward causation.
